# Supplementary material for: The interactome of intact mitochondria by cross-linking mass spectrometry provides evidence for coexisting respiratory supercomplexes
Source: Mol Cell Proteomics. 2017 Dec 8;17(2):216–32. doi: 10.1074/mcp.RA117.000470 (PMC5795388; doi:10.1074/mcp.RA117.000470)
Supplement: Supplemental Data [file supp_RA117.000470_133922_0_supp_23978_fzffwf.zip › spectra_annotation/mito_spectra_annotation/261-1-13-1-10-1.pdf]

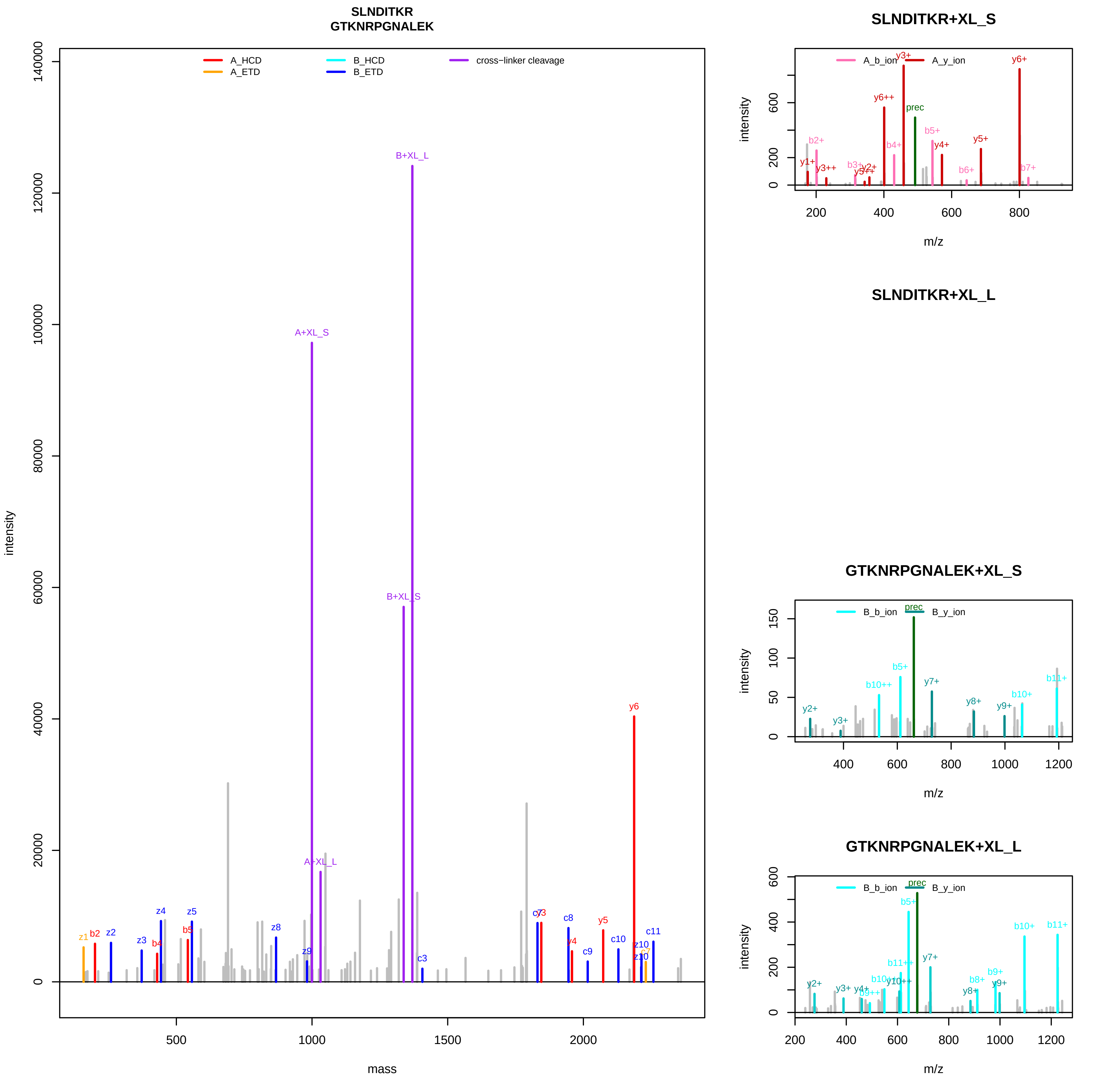
SLNDITKR
GTKNRPGNALEK
A_HCD
A_ETD
B_HCD
B_ETD
cross−linker cleavage
B+XL_L
A+XL_S
B+XL_S
A+XL_L
y6
z1
b2
z2
z3
b4
z4
b5
z5
z8
z9
c3
c7
y3
c8
y4
c9
y5
c10
z10
c11
intensity
mass
0
20000
40000
60000
80000
100000
120000
140000
500
1000
1500
2000
SLNDITKR+XL_S
A_b_ion
A_y_ion
y1+
b2+
y3++
b3+
y5++
y2+
y6++
y3+
b4+
prec
b5+
y4+
b6+
y5+
y6+
b7+
m/z
SLNDITKR+XL_L
GTKNRPGNALEK+XL_S
B_b_ion
B_y_ion
y2+
y3+
b10++
b5+
y7+
y8+
y9+
b10+
b11+
GTKNRPGNALEK+XL_L
y4+
b9++
b10++
b11++
y10++
y8+
b8+
b9+
y9+
